# Supplementary material for: Discovery and application of insertion-deletion (INDEL) polymorphisms for QTL mapping of early life-history traits in Atlantic salmon
Source: BMC Genomics. 2010 Mar 8;11:156. doi: 10.1186/1471-2164-11-156 (PMC2838853; doi:10.1186/1471-2164-11-156)
Supplement: Additional file 2 — Information on developed 76 locus single-run INDEL panel in Atlantic salmon. Information on fluorescence labeling, primer concentrations, PCR pooling and links to alignments, INDEL motifs and GENESCAN (Burge and Karlin 1997) predictions of genes/exons are available in html format. [file 1471-2164-11-156-S2.ZIP › Additionalfile2/table10259.html]

Contig Alignment

```
Base	A B C D E F G H I J K L M N O P Q   
1	A A . . . A . . . . . . . . . . .    
2	G G . . . G . . . . . . . . . . .    
3	A A . . . A . . . . . . . . . . .    
4	G G . . . G . . . . . . . . . . .    
5	T T . . . T . . . . . . . . . . .    
6	T T . . . T . . . . . . . . . . .    
7	G G G . . G . G . . . . . . . . .    
8	A A A . . A . A . . . . . . . . .    
9	C C C . C C . C C . . . . . . . .    
10	T T T . T T . T T . . . . . . . .    
11	T T T . T T . T T . . . . . . . .    
12	C C C . C C . C C . . . . . . . .    
13	T T T . T T . T T . . . . . . . .    
14	G G G . G G . G G . . . . . . . .    
15	A A A . A A . A A . . . . . . . .    
16	A A A . A A . A A . . . . . . . .    
17	T T T . T T . T T . . . . . . . .    
18	T T T . T T . T T . . . . . . . .    
19	T T T . T T . T T . . . . . . . .    
20	T T T . T T . T T . . . . . . . .    
21	A A A . A A . A A . . . . . . . .    
22	T T T . T T . T T . . . . . . . .    
23	T T T . T T . T T . . . . . . . .    
24	T T T . T T . T T . . . . . . . .    
25	T T T . T T . T T . . . . . . . .    
26	G G G . G G . G G . . . . . . . .    
27	T T T . T T . T T . . . . . . . .    
28	G G G . G G . G G . . . . . . . .    
29	T T T . T T . T T . . . . . . . .    
30	T T T . T T . T T . . . . . . . .    
31	A A A . A A . A A . . . . . . . .    
32	A A A . A A . A A . . . . . . . .    
33	T T T . T T . T T . . . . . . . .    
34	A A A . A A . A A . . . . . . . .    
35	C C C . C C . C C . . . . . . . .    
36	A A A . A A . A A . . . . . . . .    
37	A A A . A A . A A . . . . . . . .    
38	T T T . T T . T T . . . . . . . .    
39	A A A . A A . A A . . . . . . . .    
40	A A A . A A . A A . . . . . . . .    
41	A A A . A A . A A . . . . . . . .    
42	T T T . T T . T T . . . . . . . .    
43	A A A . A A . A A . . . . . . . .    
44	T T T . T T . T T . . . . . . . .    
45	A A A . A A . A A . . . . . . . .    
46	C C C . C C . C C . . . . . . . .    
47	A A A . A A . A A . . . . . . . .    
48	A A A . A A . A A . . . . . . . .    
49	A A A . A A . A A . . . . . . . .    
50	A A A . A A . A A . . . . . . . .    
51	C C C . C C . C C . . . . . . . .    
52	T T T . T T . T T . . . . . . . .    
53	T T T . T T . T T . . . . . . . .    
54	A A A . A A . A A . . . . . . . .    
55	A A A . A A . A A . . . . . . . .    
56	A A A . A A . A A . . . . . . . .    
57	C C C . C C . C C . . . . . . . .    
58	T T T . T T . T T . . . . . . . .    
59	A A A . A A . A A . . . . . . . .    
60	C C C . C C . C C . . . . . . . .    
61	T T T . T T . T T . . . . . . . .    
62	C C C . C C . C C . . . . . . . .    
63	A A A . A A . A A . . . . . . . .    
64	A A A . A A . A A . . . . . . . .    
65	C C C . C C . C C . . . . . . . .    
66	A A A . A A . A A . . . . . . . .    
67	T T T . T T . T T . . . . . . . .    
68	G G G . G G . G G . . . . . . . .    
69	C C C . C C . C C . . . . . . . .    
70	G G G . G G . G G . . . . . . . .    
71	A A A . A A . A A . . . . . . . .    
72	C C C . C C . C C . . . . . . . .    
73	T T T . T T . T T . . . . . . . .    
74	A A A . A A . A A . . . . . . . .    
75	A A A . A A . A A . . . . . . . .    
76	A A A . A A . A A . . . . . . . .    
77	A A A . A A . A A . . . . . . . .    
78	A A A . A A . A A . . . . . . . .    
79	G G G . G G . G G . . . . . . . .    
80	G G G . G G . G G . . . . . . . .    
81	A A A . A A . A A . . . . . . . .    
82	G G G . G G . G G . . . . . . . .    
83	A A A . A A . A A . . . . . . . .    
84	G G G . G G . G G . . . . . . . .    
85	G G G . G G . G G . . . . . . . .    
86	A A A . A A . A A . . . . . . . .    
87	G G G . G G . G G . . . . . . . .    
88	A A A . A A . A A . . . . . . . .    
89	G G G . G G . G G . . . . . . . .    
90	T T T . T T . T T . . . . . . . .    
91	T T T . T T . T T . . . . . . . .    
92	T T T . T T . T T . . . . . . . .    
93	C C C . C C . C C . . . . . . . .    
94	T T T . T T . T T . . . . . . . .    
95	G G G . G G . G G . . . . . . . .    
96	T T T . T T . T T . . . . . . . .    
97	A A A . A A . A A . . . . . . . .    
98	C C C . C C . C C . . . . . . . .    
99	A A A . A A . A A . . . . . . . .    
100	G G G . G G . G G . . . . . . . .    
101	T T T . T T . T C . . . . . . . .    
102	A A A . A A . A A . . . . . . . .    
103	C C C . C C . C C . . . . . . . .    
104	A A A . A A . A A . . . . . . . .    
105	A A A . A A . A A . . . . . . . .    
106	T T T . T T . T T . . . . . . . .    
107	G G G . G G . G G . . . . . . . .    
108	A A A . A A . A A . . . . . . . .    
109	A A A . A A . A A . . . . . . . .    
110	A A A . A A . A A . . . . . . . .    
111	T T T . T T . T T . . . . . . . .    
112	T T T . T T . T T . . . . . . . .    
113	C C C . C C . C C . . . . . . . .    
114	A A A . A T . A A . . . . . . . .    
115	G G G . G G . G G . . . . . . . .    
116	G G G . G G . G G . . . . . . . .    
117	A A A . A A . A A . . . . . . . .    
118	T T T . T T . T T . . . . . . . .    
119	A A A . A A . A A . . . . . . . .    
120	C C C . C C . C C . . . . . . . .    
121	C C C . C C . C C . . . . . . . .    
122	A A A . A A . A A . . . . . . . .    
123	G G G . G G . G G . . . . . . . .    
124	C C C . C C . C C . . . . . . . .    
125	C C C . C C . C C . . . . . . . .    
126	G G G . G G . G G . . . . . . . .    
127	A A A . A A . A A . . . . . . . .    
128	C C C . C C . C C . . . . . . . .    
129	A A A . A A . A A . . . . . . . .    
130	A A A . A A . A A . . . . . . . .    
131	G G G . G G . G G . . . . . . . .    
132	T T T . T T . T T . . . . . . . .    
133	T T T . T T . T T . . . . . . . .    
134	G G G . G G . G G . . . . . . . .    
135	T T T . T T . T T . . . . . . . .    
136	C C C . C C . C C . . . . . . . .    
137	A A A . A A . A A . . . . . . . .    
138	A A A . A A . A A . . . . . . . .    
139	A A A . A A . A A . . . . . . . .    
140	C C C . C C . C C . . . . . . . .    
141	T T T . T T . T T . . . . . . . .    
142	T T T . T T . T T . . . . . . . .    
143	T T T . T T . T T . . . . . . . .    
144	A A A . A A . A A . . . . . . . .    
145	C C C . C C . C C . . . . . . . .    
146	A A A . A A . A A . . . . . . . .    
147	A A A . A A . A A . . . . . . . .    
148	A A A . A A . A A . . . . . . . .    
149	A A A . A A . A A . . . . . . . .    
150	T T T . T T . T T . . . . . . . .    
151	C C C . C C . C C . . . . . . . .    
152	T T T . T T . T T . . . . . . . .    
153	A A A . A A . A A . . . . . . . .    
154	C C C . C C . C C . . . . . . . .    
155	A A A . A A . A A . . . . . . . .    
156	A A A . A A . A A . . . . . . . .    
157	A A A . A A . A A . . . . . . . .    
158	C C C . C C . C C . . . . . . . .    
159	A A A . A A . A A . . . . . . . .    
160	T T T . T T . T T . . . . . . . .    
161	C C C . C C . C C . . . . . . . .    
162	C C C . C C . C C . . . . . . . .    
163	C C C . C C . C C . . . . . . . .    
164	T T T . T T . T T . . . . . . . .    
165	T T T . T T . T T . . . . . . . .    
166	A A A . A A . A A . . . . . . . .    
167	A A A . A A . A A . . . . . . . .    
168	A A A . A A . A A . . . . . . . .    
169	G G G . G G . G G . . . . . . . .    
170	T T T . T T . T T . . . . . . . .    
171	T T T . T T . T T . . . . . . . .    
172	T T T . T T . T T . . . . . . . .    
173	G G G . G G . G G . . . . . . . .    
174	C C C . C C . C C . . . . . . . .    
175	T T T . T T . T T . . . . . . . .    
176	T T T . T T . T T . . . . . . . .    
177	A A A . A A . A A . . . . . . . .    
178	A A A . A A . A A . . . . . . . .    
179	T T T . T T . T T . . . . . . . .    
180	T T T . T T . T T . . . . . . . .    
181	T T T . T T . T T . . . . . . . .    
182	T T T . T T . T T . . . . . . . .    
183	T T T . T T . T T . . . . . . . .    
184	T T T . T T . T T . . . . . . . .    
185	A A A . A A A A A . . A . . . . .    
186	A A A . A A A A A . . A . . . . .    
187	A A A . A A A A A . . A . . . . .    
188	T T T . T T T T T . . T . . . . .    
189	A A A . A A A A A . . A . . . . .    
190	A A A . A A A A A . . A . . . . .    
191	C C C . C C C C C . . C . . . . .    
192	T T T . T T T T T . . T . . . . .    
193	T T T . T T T T T . . T . . . . .    
194	C C C . C C C C C . . C . . . . .    
195	A A A . A A A A A . A A . . . . .    
196	A A A . A A A A A . A A . . . . .    
197	T T T . T T T T T . T T . . . . .    
198	T T T . T T T T T . T T . . . . .    
199	T T T . T T T T T . T T . . . . .    
200	A A A . A A A A A . A A . . . . .    
201	T T T . T T T T T . T T . . . . .    
202	T T T . T T T T T . T T . . . . .    
203	C C C . C C C C C . C C . . . . .    
204	C C C . C C C C C . C C . . . . .    
205	A A A . A A A A A . A A . . . . .    
206	T T T . T T T T T . T T . . . . .    
207	T T T . T T T T T . T T . . . . .    
208	G G G . G G G G G . G G . G . . .    
209	T T T . T T T T T . T T . T . . .    
210	C C C . C C C C C . C C . C . . .    
211	A A A . A A A A A . A A . A . . .    
212	G G G . G G G G G . G G . G . . .    
213	A A A . A A A A A . A A . A . . .    
214	G G G . G G G G G . G G . G . . .    
215	T T T . T T T T T . T T . T . . .    
216	T T T . T T T T T . T T . T . . .    
217	G G G . G G G G G . G G . G . . .    
218	A A A . A A A A A . A A . A . . .    
219	G G G . G G G G G . G G . G . . .    
220	C C C . C C C C C . C C . C . . .    
221	A A A . A A A A A . A A . A . . .    
222	A A A . A A A A A . A A . A . . .    
223	G G G . G G G G G . G G . G . . .    
224	A A A . A A A A A . A A . A . . .    
225	C C C . C C C C C . C C . C . . .    
226	A A A . A A A A A . A A . A . . .    
227	A A A . A A A A A . A A . A . . .    
228	A A A . A A A A A . A A . A . . .    
229	- - - . - - - A - . - - . - . . .    
230	T T T . T T T T T . T T . T . . .    
231	G G G . G G G G G . G G . G . . .    
232	T T T . T T T T T . T T . T . . .    
233	C C C . C C C C C . C C . C . . .    
234	A A A . A A A A A . A A . A . . .    
235	T T T . T T T T T . T T . T . . .    
236	A A A . A A A A A . A A . A . . .    
237	T T T . T T T T T . T T . T . . .    
238	T T T . T T T T T . T T . T . . .    
239	C C C . C C C C C . C C . C . . .    
240	A A A . A A A A A . A A . A . . .    
241	T T T . T T T T T . T T . T . . .    
242	T T T . T T T T T . T T . T . . .    
243	C C C . C C C C C . C C . C . . .    
244	T T T . T T T T T . T T . T . . .    
245	C C C . C C C C C . C C . C . . .    
246	A A A . A A A A A . A A . A . . .    
247	G G G . G G G G G . G G . G . . .    
248	T T T . T T T T T . T T . T . . .    
249	A A A . A A A A A . A A . A . . .    
250	T T T . T T T T T T T T . T . . .    
251	T T T . T T T T T T T T . T . . .    
252	T T T . T T T T T T T T . T . . .    
253	T T T . T T T T T T T T . T . . .    
254	A A A . A A A A A A A A . A . . .    
255	C C C . C C C C C C C C . C . . .    
256	T T T . T T T T T T T T . T . . .    
257	A A A . A A A A A A A A . A . . .    
258	T T T . T T T T T T T T . T . . .    
259	A A A . A A A A A A A A . A . . .    
260	T T T . T T T T T T T T . T . . .    
261	A A A . A A A A A A A A . A . . .    
262	C C C . C C C C C C C C . C . . .    
263	T T T . T T T T T T T T . T . . .    
264	T T T . T T T T T T T T . T . . .    
265	G G G . G G G G G G G G . G . . .    
266	A A A A A A A A A A A A . A . . .    
267	T T T T T T T T T T T T . T . . .    
268	A A A A A A A A A A A A . A . . .    
269	C C C C C C C C C C C C . C . . .    
270	A A A A A A A A A A A A . A . . .    
271	T T T T T T T T T T T T . T . . .    
272	T T T T T T T T T T T T . T . . .    
273	G G G G G G G G G G G G . G . . .    
274	A A A A A A A A A A A A . A . . .    
275	T T T T T T T T T T T T . T . . .    
276	T T T T T T T T T T T T . T . . .    
277	A A A A A A A A A A A A . A . . .    
278	A A A A A A A A A A A A . A . . .    
279	A A A A A A A A A A A A . A . . .    
280	A A A A A A A A A A A A . A . . .    
281	A A A A A A A A A A A A . A . . .    
282	G G G G G G G G G G G G . G . . .    
283	C C C C C C C C C C C C . C . . .    
284	A A A A A A A A A A A A . A . . .    
285	C C C C C C C C C C C C . C . . .    
286	A A A A A A A A A A A A . A . . .    
287	A A A A A A A A A A A A . A . . .    
288	G G G G G G G G G G G G . G . . .    
289	T T T T T T T T T T T T . T . . .    
290	T T T T T T T T T T T T . T . . .    
291	A A A A A A A A A A A A . A . . .    
292	A A A A A A A A A A A A . A . . .    
293	A A A A A A A A A A A A . A . . .    
294	G G G G G G G G G G G G . G . . .    
295	C C C C C C C C C C C C . C . . .    
296	C C C C C C C C C C C C . C . . .    
297	A A A A A A A A A A A A . A . . .    
298	C C C C C C C C C C C C . C . . .    
299	A A A A A A A A A A A A . A . . .    
300	G G G G G G G G G G G G . G . . .    
301	T T T T T T T T T T T T . T . . .    
302	C C C C C C C C C C C C . C . . .    
303	C C C C C C C C C C C C . C . . .    
304	A A A A A A A A A A A A . A . . .    
305	T T T T T T T T T T T T . T . . .    
306	C C C C C C C C C C C C . C . . .    
307	T T T T T T T T T T T T . T . . .    
308	C C C C C C C C C C C C . C . . .    
309	A A A A A A A A A A A A . A . . .    
310	A A A A A A A A A A A A . A . . .    
311	A A A A A A A A A A A A . A . . .    
312	A A A A A A A A A A A A . A . . .    
313	C C C C C C C C C C C C . C . . .    
314	A A A A A A A A A A A A . A . . .    
315	C C C C C C C C C C C C . C . . .    
316	A A A A A A A A A A A A . A . . .    
317	A A A A A G A A A A A A . A . . .    
318	A A A A A A A A A A A A . A . . .    
319	C C C C C C C C C C C C . C . . .    
320	A A A A A A A A A A A A . A . . .    
321	T T T T T T T T T T T T . T . . .    
322	C C C C C C C C C C C C . C . . .    
323	G G G G G G G G G G G G . G . . .    
324	C C C C C C C C C C C C . C . . .    
325	T T T T T T T T T T T T . T . . .    
326	T T T T T T T T T T T T . T . . .    
327	A A A A A A A A A A A A . A . . .    
328	G G G G G G G G G G G G . G . . .    
329	T T T T T T T T T T T T . T . . .    
330	T T T T T T T T T T T T . T . . .    
331	T T T T T T T T T T T T . T . . .    
332	C C C C C C C C C C C C . C . . .    
333	A A A A A A A A A A A A . A . . .    
334	A A A A A A A A A A A A . A . . .    
335	C C C C C C C C C C C C . C . . .    
336	T T T T T T T T T T T T . T . . .    
337	A A A A A A A A A A A A . A . . .    
338	C C C C C C C C C C C C . C . . .    
339	T T T T T T T T T T T T . T . . .    
340	G G G G G G G G G G G G . G . . .    
341	A A A A A A A A A A A A . A . . .    
342	C C C C C C C C C C C C . C . . .    
343	T T T T T T T T T T T T . T . . .    
344	T T T T T T T T T T T T . T . . .    
345	T T T T T T T T T T T T . T . . .    
346	C C C C C C C C C C C C . C . . .    
347	T T T T T T T T T T T T . T . . .    
348	C C C C C C C C C C C C . C . . .    
349	A A A A A A A A A A A A . A . . .    
350	C C C C C C C C C C C C . C . . .    
351	A A A A A A A A A A A A . A . . .    
352	T T T T T T T T T T T T . T . . .    
353	T T T T T T T T T T T T . T . . .    
354	T T T T T T T T T T T T . T . . .    
355	C C C C C C C C C C C C C C . . .    
356	C C C C C C C C C C C C C C . . .    
357	C C C C C C C C C C C C C C . . .    
358	A A A A A A A A A A A A A A . . .    
359	G G G G G G G G G G G G G G . . .    
360	T T T T T T T T T T T T T T . . .    
361	T T T T T T T T T T T T T T . . .    
362	G G G G G G G G G G G G G G . . .    
363	A A A A A A A A A A A A A A . . .    
364	T T T T T T T T T T T T T T . . .    
365	G G G G G G G G G G G G G G . . .    
366	C C C C C C C C C C C C C C . . .    
367	A A A A A A A A A A A A A A . . .    
368	T T T T T T T T T T T T T T . . .    
369	T T T T T T T T T T T T T T . . .    
370	T T T T T T T T T T T T T T . . .    
371	T T T T T T T T T T T T T T . . .    
372	T T T T T T T T T T T T T T . . .    
373	T T T T T T T T T T T T T T . . .    
374	T T T T T T T T T T T T T T . . .    
375	T T T T T T T T T T T T T T . . .    
376	T T T T T T T T T T T T T T . . .    
377	T T T T T T T T T T T T T T . . .    
378	- - - - - - T T - T - T - - . . .    
379	A A A A A A A A A A A A A A . . .    
380	T T T T T T T T T T T T T T . . .    
381	T T T T T T T T T T T T T T . . .    
382	G G G G G G G G G G G G G G . . .    
383	T T T T T T T T T T T T T T . . .    
384	T T T T T T T T T T T T T T . . .    
385	T T T T T T T T T T T T T T . . .    
386	T T T T T T T T T T T T T T . . .    
387	C C C C C C C C C C C C C C . . .    
388	A A A A A A A A A A A A A A . . .    
389	A A A A A A A A A A A A A A . . .    
390	G G G G G G G G G G G G G G . . .    
391	C C C C C C C C C C C C C C . . .    
392	C C C C C C C C C C C C C C . . .    
393	A A A A A A A A A A A A A A . . .    
394	A A A A A A A A A A A A A A . . .    
395	C C C C C C C C C C C C C C . . .    
396	A A A A A A A A A A A A A A . . .    
397	C C C C C C C C C C C C C C . . .    
398	A A A A A A A A A A A A A A . . .    
399	T T T T T T T T T T T T T T . . .    
400	A A A A A A A A A A A A A A . . .    
401	T T T T T T T T T T T T T T . . .    
402	T T T T T T T T T T T T T T . . .    
403	T T T T T T T T T T T T T T . . .    
404	A A A A A A A A A A A A A A . . .    
405	A A A A A A A A A A A A A A . . .    
406	C C C C C C C C C C C C C C . . .    
407	A A A A A A A A A A A A A A . . .    
408	G G G G G G G G G G G G G G . . .    
409	G G G G G G G G G G G G G G . . .    
410	A A A A A A A A A A A A A A . . .    
411	A A A A A A A A A A A A A A . . .    
412	C C C C C C C C C C C C C C . . .    
413	A A A A A A A A A A A A A A . . .    
414	T T T T T T T T T T T T T T . . .    
415	T T T T T T T T T T T T T T . . .    
416	C C C C C C C C C C C C C C . . .    
417	A A A A A A A A A A A A A A . . .    
418	G G G G G G G G G G G G G G . . .    
419	G G G G G G G G G G G G G G . . .    
420	T T T T T T G T T T T T T T . . .    
421	G G G G G G G G G G G G G G . . .    
422	C C C C C C C C C C C C C C . . .    
423	C C C C C C C C C C C C C C . . .    
424	A A A A A A A A A A A A A A . . .    
425	. T T T T T T T T T T T T T . . .    
426	. T T T T T T T T T T T T T . . .    
427	. C C C C C C C C C C C C C . . .    
428	. G G G G G G G G G G G G G . . .    
429	. T T T T T T T T T T T T T . . .    
430	. T T T T T T T T T T T T T . . .    
431	. C C C C C C C C C C C C C . . .    
432	. A A A A A A A A A A A A A . . .    
433	. G G G G G G G G G G G G G . . .    
434	. A A A A A A A A A A A A A . . .    
435	. T T T T T T T T T T T T T . . .    
436	. T T T T T T T T T T T T T . . .    
437	. T T T T T T T T T T T T T . . .    
438	. T T T T T T T T T T T T T . . .    
439	. C C C C C C C C C C C C C . . .    
440	. A A A A A A A A A A A A A . . .    
441	. A A A A A A A A A A A A A . . .    
442	. A A A A A A A A A A A A A . . .    
443	. A A A A A A A A A A A A A . . .    
444	. G G G G G G G G G G G G G . . .    
445	. A A A A A A A A A A A A A . . .    
446	. T T T T T T T T T T T T T . . .    
447	. G G G G G G G G G G G G G . . .    
448	. T T T T T T T T T T T T T . . .    
449	. A A A A A A A A A A A A A . . .    
450	. G G G G G G G G G G G G G . . .    
451	. A A A A A A A A A A A A A . . .    
452	. A A A A A A A A A A A A A . . .    
453	. T T T T T T T T T T T T T . . .    
454	. A A A A A A A A A A A A A . . .    
455	. A A A A A A A A A A A A A . . .    
456	. C C C C C C C C C C C C C . . .    
457	. T T T T T T T T T T T T T . . .    
458	. T T T T T T T T T T T T T . . .    
459	. A A A A A A A A A A A A A . . .    
460	. A A A A A A A A A A A A A . . .    
461	. A A A A A A A A A A A A A . . .    
462	. G G G G G G G G G G G G G . . .    
463	. T T T T T T T T T T T T T . . .    
464	. A A A A A A A A A A A A A . . .    
465	. G G G G G G G G G G G G G . . .    
466	. C C C C C C C C C C C C C . . .    
467	. T T T T T T T T T T T T T . . .    
468	. C C C C C C C C C C C C C . . .    
469	. A A A A A A A A A A A A A . . .    
470	. G G G G G G G G G G G G G . . .    
471	. C C C C C C C C C C C C C . . .    
472	. A A A A A A A A A A A A A . . .    
473	. C C C C C C C C C C C C C . . .    
474	. A A A A A A A A A A A A A . . .    
475	. C C C C C C C C C C C C C . . .    
476	. A A A A A A A A A A A A A . . .    
477	. C C C C C C C C C C C C C . . .    
478	. A A A A A A A A A A A A A . . .    
479	. C C C C C C C C C C C C C . . .    
480	. A A A A A A A A A A A A A . . .    
481	. T T T T T T T T T T T T T . . .    
482	. T T T T T T T T T T T T T . . .    
483	. C C C C C C C C C C C C C . . .    
484	. A A A A A A A A A A A A A . . .    
485	. C C C C C C C C C C C C C . . .    
486	. T T T T T T T T T T T T T . . .    
487	. C C C C C C C C C C C C C . . .    
488	. A A A A A A A A A A A A A . . .    
489	. T T T T T T T T T T T T T . . .    
490	. A A A A A A A A A A A A A . . .    
491	. C C C C C C C C C C C C C . . .    
492	. T T T T T T T T T T T T T . . .    
493	. C C C C C C C C C C C C C . . .    
494	. A A A A A A A A A A A A A . . .    
495	. A A A A A A A A A A A A A . . .    
496	. G G G G G G G G G G G G G . . .    
497	. A A A A A A A A A A A A A . . .    
498	. C C C C C C C C C C C C C . . .    
499	. C C C C C C C C C C C C C . . .    
500	. A A A A A A A A A A A A A . . .    
501	. G G G G G G G G G G G G G . . .    
502	. A G A A G A A G A A A A G . . .    
503	. G G G G G G G G G G G G G . . .    
504	. A A A A A A A A A A A A A . . .    
505	. G G G G G G G G G G G G G . . .    
506	. A A A A A A A A A A A A A . . .    
507	. T T T T T T T T T T T T T . . .    
508	. G G G G G G G G G G G G G . . .    
509	. A A A A A A A A A A A A A . . .    
510	. A A A A A A A A A A A A A . . .    
511	. T T T T T T T T T T T T T . . .    
512	. G G G G G G G G G G G G G . . .    
513	. A A A A A A A A A A A A A . . .    
514	. C C C C C C C C C C C C C . . .    
515	. T T T T T T T T T T T T T . . .    
516	. A A A A A A A A A A A A A . . .    
517	. T T T T T T T T T T T T T . . .    
518	. T T T T T T T T T T T T T . . .    
519	. A A A A A A A A A A A A A . . .    
520	. G G G G G G G G G G G G G . . .    
521	. A A A A A A A A A A A A A . . .    
522	. C C C C C C C C C C C C C . . .    
523	. T T T T T T T T T T T T T . . .    
524	. A A A A A A A A A A A A A . . .    
525	. T T T T T T T T T T T T T . . .    
526	. T T T T T T T T T T T T T . . .    
527	. A A A A A A A A A A A A A . . .    
528	. A - - - - A A - A A A - - . . .  6	 indel  
529	. G - - - - G G - G G G - - . . .  6	 indel  
530	. G - - - - G G - G G G - - . . .  6	 indel  
531	. A - - - - A A - A A A - - . . .  6	 indel  
532	. A - - - - A A - A A A - - . . .  6	 indel  
533	. A A A A A A A A A A A A A . . .    
534	. G G G G G G G G G G G G G . . .    
535	. T T T T T T T T T T T T T . . .    
536	. A A A A A A A A A A A A A . . .    
537	. T T T T T T T T T T T T T . . .    
538	. A A A A A A A A A A A A A . . .    
539	. A A A A A A A A A A A A A . . .    
540	. A A A A A A A A A A A A A . . .    
541	. A A A A A A A A A A A A A . . .    
542	. A A A A A A A A A A A A A . . .    
543	. G G G G G G G G G G G G G . . .    
544	. G G G G G G G G G G G G G . . .    
545	. A A A A A A A A A A A A A . . .    
546	. G G G G G G G G G G G G G . . .    
547	. G G G G G G G G G G G G G . . .    
548	. A A A A A A A A A A A A A . . .    
549	. A A A A A A A A A A A A A . . .    
550	. A A A A A A A A A A A A A . . .    
551	. C C C C C C C C C C C C C . . .    
552	. T T T T T T T T T T T T T . . .    
553	. C C C C C C C C C C C C C . . .    
554	. A A A A A A A A A A A A A . . .    
555	. T T T T T T T T T T T T T . . .    
556	. G G G G G G G G G G G G G . . .    
557	. A A A A A A A A A A A A A . . .    
558	. G G G G G G G G G G G G G . . .    
559	. G G G G G G G G G G G G G . . .    
560	. A A A A A A A A A A A A A . . .    
561	. T T T T T T T T T T T T T . . .    
562	. G G G G G G G G G G G G G . . .    
563	. T T T T T T T T T T T T T . . .    
564	. C C C C C C C C C C C C C . . .    
565	. A A A A A A A A A A A A A . . .    
566	. G G G G G G G G G G G G G . . .    
567	. C C C C C C C C C C C C C . . .    
568	. C C C C C C C C C C C C C . . .    
569	. T T T T T T T T T T T T T . . .    
570	. G G G G G G G G G G G G G . . .    
571	. T T T T T T T T T T T T T . . .    
572	. C C C C C C C C C C C C C . . .    
573	. T T T T T T T T T T T T T . . .    
574	. C C C C C C C C C C C C C . . .    
575	. T T T T T T T T T T T T T . . .    
576	. G G G G G G G G G G G G G . . .    
577	. C C C C C C C C C C C C C . . .    
578	. C C C C C C C C C C C C C . . .    
579	. C C C C C C C C C C C C C . . .    
580	. A A A A A A A A A A A A A . . .    
581	. C C C C C C C C C C C C C . . .    
582	. A A A A A A A A A A A A A . . .    
583	. T T T T T T T T T T T T T . . .    
584	. C C C C C C C C C C C C C . . .    
585	. A A A A A A A A A A A A A . . .    
586	. G G G G G G G G G G G G G . . .    
587	. T T T T T T T T T T T T T . . .    
588	. G G G G G G G G G G G G G . . .    
589	. T T T T T T T T T T T T T . . .    
590	. T T T T T T T T T T T T T . . .    
591	. C C C C C C C C C C C C C . . .    
592	. A A A A A A A A A A A A A . . .    
593	. A A A A A A A A A A A A A . . .    
594	. C C C C C C C C C C C C C . . .    
595	. T T T T T T T T T T T T T . . .    
596	. G G G G G G G G G G G G G . . .    
597	. A A A A A A A A A A A A A . . .    
598	. A A A A A A A A A A A A A . . .    
599	. C C C C C C C C C C C C C . . .    
600	. A A A A A A A A A A A A A . . .    
601	. A A A A A A A A A A A A A . . .    
602	. C C C C C C C C C C C C C . . .    
603	. C C C C C C C C C C C C C . . .    
604	. A A A A A A A A A A A A A . . .    
605	. G G G G G G G G G G G G G . . .    
606	. T T T T T T T T T T T T T . . .    
607	. T T T T T T T T T T T T T . . .    
608	. A A A A A A A A A A A A A . . .    
609	. T T T T T T T T T T T T T . . .    
610	. C C C C C C C C C C C C C . . .    
611	. C C C C C C C C C C C C C . . .    
612	. T T T T T T T T T T T T T . . .    
613	. T T T T T T T T T T T T T . . .    
614	. G G G G G G G G G G G G G . . .    
615	. T T T T T T T T T T T T T . . .    
616	. T T T T T T T T T T T T T . . .    
617	. A A A A A A A A A A A A A . . .    
618	. A A A A A A A A A A A A A . . .    
619	. G G G G G G G G G G G G G . . .    
620	. A A A A A A A A A A A A A . . .    
621	. C C C C C C C C C C C C C . . .    
622	. T T T T T T T T T T T T T . . .    
623	. T T T T T T T T T T T T T . . .    
624	. C C C C C C C C C C C C C . . .    
625	. A A A A A A A A A A A A A . . .    
626	. C C C C C C C C C C C C C . . .    
627	. T T T T T T T T T T T T T . . .    
628	. T T T T T T T T T T T T T . . .    
629	. T T T T T T T T T T T T T . . .    
630	. G G G G G G G G G G G G G . . .    
631	. A A A A A A A A A A A A A . . .    
632	. C C C C C C C C C C C C C C . .    
633	. C C C C C C C C C C C C C C . .    
634	. G G G G G G G G G G G G G G . .    
635	. A A A A A A A A A A A A A A . .    
636	. C C C C C C C C C C C C C C . .    
637	. A A A A A A A A A A A A A A . .    
638	. C C C C C C C C C C C C C C . .    
639	. C C C C C C C C C C C C C C . .    
640	. C C C C C C C C C C C C C C . .    
641	. C C C C C C C C C C C C C C . .    
642	. A A A A A A A A A A A A A A . .    
643	. C C C C C C C C C C C C C C . .    
644	. A A A A A A A A A A A A A A . .    
645	. A A A A A A A A A A A A A A . .    
646	. C C C C C C C C C C C C C C . .    
647	. A A A A A A A A A A A A A A . .    
648	. C C C C C C C C C C C C C C . .    
649	. A A A A A A A A A A A A A A . .    
650	. C C C C C C C C C C C C C C . .    
651	. A A A A A G A A G A A A A A . .    
652	. C C C C C C C C C C C C C C . .    
653	. A A A A A A A A A A A A A A . .    
654	. T T T T T T T T T T T T T T . .    
655	. C C C C C C C C C C C C C C . .    
656	. C C C C C C C C C C C C C C . .    
657	. A A A A A A A A A A A A A A . .    
658	. T T T T T T T T T T T T T T . .    
659	. G G G G G G G G G G G G G G . .    
660	. A A A A A A A A A A A A A A . .    
661	. T T T T T T T T T T T T T T . .    
662	. . A A A A A A A A A A A A A . .    
663	. . T T T T T T T T T T T T T . .    
664	. . T T T T T T T T T T T T T . .    
665	. . A A A A A A A A A A A A A . .    
666	. . C C C C C C C C C C C C C . .    
667	. . A A A A A A A A A A A A A . .    
668	. . G G G G G G G G G G G G G . .    
669	. . C C C C C C C C C C C C C . .    
670	. . A A A A A A A A A A A A A . .    
671	. . C C C C C C C C C C C C C . .    
672	. . A A A A A A A A A A A A A . .    
673	. . C C C C C C C C C C C C C . .    
674	. . A A A A A A A A A A A A A . .    
675	. . T T T T T T T T T T T T T . .    
676	. . G G G G G G G G G G G G G . .    
677	. . G G G G G G G G G G G G G . .    
678	. . A A A A A A A A A A A A A . .    
679	. . C C C C C C C C C C C C C . .    
680	. . A A A A A A A A A A A A A . A    
681	. . C C . C C C C C C C C C C . C    
682	. . A A . A C A A A A A A A A A A    
683	. . . C . C C C C C C C C C C C C    
684	. . . A . A A A A A A A A A A A A    
685	. . . C . C C C C C C C C C C C C    
686	. . . A . A A A A A A A A A A A A    
687	. . . C . C C C C C C C C C C C C    
688	. . . A . A A A A A A A A A A A A    
689	. . . C . C C C C C C C C C C C C    
690	. . . C . C C C C C C C C C C C C    
691	. . . . . C C C C C C C C C C C C    
692	. . . . . C C C C C C C C C C C C    
693	. . . . . T T T T T T T T T T T T    
694	. . . . . C C C C C C C C C C C C    
695	. . . . . A A A A A A A A A A A A    
696	. . . . . A A A A A A A A A A A A    
697	. . . . . A A A A A A A A A A A A    
698	. . . . . C C C C C C C C C C C C    
699	. . . . . A A A A A A A A A A A A    
700	. . . . . T T T T T T T T T T T T    
701	. . . . . T T T T T T T T T T T T    
702	. . . . . C C C C C C C C C C C C    
703	. . . . . C C C C C C C C C C C C    
704	. . . . . C C C C C C C C C C C C    
705	. . . . . A A A A A A A A A A A A    
706	. . . . . A A A A A A A A A A A A    
707	. . . . . C C C C C C C C C C C C    
708	. . . . . C C C C C C C C C C C C    
709	. . . . . C C C C C C C C C C C C    
710	. . . . . C C C C C C C C C C C C    
711	. . . . . T T T T T T T T T T T T    
712	. . . . . C C C C C C C C C C C C    
713	. . . . . T T T T T T T T T T T T    
714	. . . . . T T T T T T T T T T T T    
715	. . . . . C C C C C C C C C C C C    
716	. . . . . A A A A A A A A A A A A    
717	. . . . . T T T T T T T T T T T T    
718	. . . . . G G G G G G G G G G G G    
719	. . . . . T T T T T T T T T T T T    
720	. . . . . C C C C C C C C C C C C    
721	. . . . . A A A A A A A A A A A A    
722	. . . . . C C C C C C C C C C C C    
723	. . . . . A A A A A A A A A A A A    
724	. . . . . T T . T T T T T T T T T    
725	. . . . . T T . T T T T T T T T T    
726	. . . . . C C . C C C C C C C C C    
727	. . . . . A A . A A A A A A A A A    
728	. . . . . A A . A A A A A A A A A    
729	. . . . . A A . A A A A A A A A A    
730	. . . . . G G . G G G G G G G G G    
731	. . . . . G G . G G G G G G G G G    
732	. . . . . C C . C C C C C C C C C    
733	. . . . . T T . T T T T T T T T T    
734	. . . . . G G . G G G G G G G G G    
735	. . . . . A A . A A A A A A A A A    
736	. . . . . A A . A A A A A A A A A    
737	. . . . . A A . A A A A A A A A A    
738	. . . . . G G . G G G G G G G G G    
739	. . . . . T T . T T T T T T T T T    
740	. . . . . C C . C C C C C C C C C    
741	. . . . . A A . A A A A A A A A A    
742	. . . . . C C . C C C C C C C C C    
743	. . . . . A A . A A G A A A A A A    
744	. . . . . G G . G G . G G G G G G    
745	. . . . . G G . G G . G G G G G G    
746	. . . . . T T . T T . T T T T T T    
747	. . . . . A A . A A . A A A A A A    
748	. . . . . G G . G G . G G G G G G    
749	. . . . . G G . G G . G G G G G G    
750	. . . . . T T . T T . T T T T T T    
751	. . . . . T T . T T . T T T T T T    
752	. . . . . C C . C C . C C C C C C    
753	. . . . . A A . A A . A A A A A A    
754	. . . . . G G . G G . G G G G G G    
755	. . . . . T T . T T . T T T T T T    
756	. . . . . T . . T T . T T T T T T    
757	. . . . . A . . A A . A A A A A A    
758	. . . . . T . . T T . T T T T T T    
759	. . . . . A . . A A . A A A A A A    
760	. . . . . A . . A C . A A A A A A    
761	. . . . . A . . A . . A A A A A A    
762	. . . . . C . . C . . C C C C C C    
763	. . . . . A . . A . . A A A A A A    
764	. . . . . C . . C . . C C C C C C    
765	. . . . . T . . T . . T T T T T T    
766	. . . . . C . . . . . C C C C C C    
767	. . . . . A . . . . . A A A A A A    
768	. . . . . A . . . . . A A A A A A    
769	. . . . . A . . . . . A A A A A A    
770	. . . . . A . . . . . A A A A A A    
771	. . . . . C . . . . . C C C C C C    
772	. . . . . A . . . . . A A A A A A    
773	. . . . . A . . . . . A A A A A A    
774	. . . . . G . . . . . G G G G G G    
775	. . . . . A . . . . . A A A A A A    
776	. . . . . C . . . . . C C C C C C    
777	. . . . . A . . . . . A A A A A A    
778	. . . . . T . . . . . T T T T T T    
779	. . . . . G . . . . . G G G G G G    
780	. . . . . . . . . . . T T T T T T    
781	. . . . . . . . . . . T T T T T T    
782	. . . . . . . . . . . A A A A A A    
783	. . . . . . . . . . . A A A A A A    
784	. . . . . . . . . . . T T T T T T    
785	. . . . . . . . . . . A A A A A A    
786	. . . . . . . . . . . A A A A A A    
787	. . . . . . . . . . . A A A A A A    
788	. . . . . . . . . . . T T T T T T    
789	. . . . . . . . . . . T T T T T T    
790	. . . . . . . . . . . G G G G G G    
791	. . . . . . . . . . . A A A A A A    
792	. . . . . . . . . . . A A A A A G    
793	. . . . . . . . . . . T T T T T T    
794	. . . . . . . . . . . C C C C C C    
795	. . . . . . . . . . . A A A A A A    
796	. . . . . . . . . . . G G G G G G    
797	. . . . . . . . . . . A A A A A A    
798	. . . . . . . . . . . A A A A A A    
799	. . . . . . . . . . . C C C C C C    
800	. . . . . . . . . . . T T T T T T    
801	. . . . . . . . . . . T T T T T T    
802	. . . . . . . . . . . A A A A A A    
803	. . . . . . . . . . . T T T T T T    
804	. . . . . . . . . . . T T T T T T    
805	. . . . . . . . . . . C C C C C C    
806	. . . . . . . . . . . A A A A A A    
807	. . . . . . . . . . . G G G G G G    
808	. . . . . . . . . . . T T T T T T    
809	. . . . . . . . . . . A A A A A A    
810	. . . . . . . . . . . C C C C C C    
811	. . . . . . . . . . . T T T T T T    
812	. . . . . . . . . . . T T T T T C    
813	. . . . . . . . . . . T T T T T T    
814	. . . . . . . . . . . T C C C C C    
815	. . . . . . . . . . . . T T T T T    
816	. . . . . . . . . . . . A A A A A    
817	. . . . . . . . . . . . A A A A A    
818	. . . . . . . . . . . . C C C C C    
819	. . . . . . . . . . . . A A A A A    
820	. . . . . . . . . . . . T T T T T    
821	. . . . . . . . . . . . G G G G G    
822	. . . . . . . . . . . . A A A A A    
823	. . . . . . . . . . . . T T T T T    
824	. . . . . . . . . . . . G G G G G    
825	. . . . . . . . . . . . A A A A A    
826	. . . . . . . . . . . . A A A A A    
827	. . . . . . . . . . . . T T T T T    
828	. . . . . . . . . . . . A A A A A    
829	. . . . . . . . . . . . C C C C C    
830	. . . . . . . . . . . . A A A A A    
831	. . . . . . . . . . . . A A A A A    
832	. . . . . . . . . . . . T T T T T    
833	. . . . . . . . . . . . C C C C C    
834	. . . . . . . . . . . . C C C C C    
835	. . . . . . . . . . . . A A A A A    
836	. . . . . . . . . . . . A A A A A    
837	. . . . . . . . . . . . C C C C C    
838	. . . . . . . . . . . . T T T T T    
839	. . . . . . . . . . . . G G G G G    
840	. . . . . . . . . . . . A A A A A    
841	. . . . . . . . . . . . G G G G G    
842	. . . . . . . . . . . . G G G G G    
843	. . . . . . . . . . . . G G G G G    
844	. . . . . . . . . . . . G G G G G    
845	. . . . . . . . . . . . A A A A A    
846	. . . . . . . . . . . . A A A A A    
847	. . . . . . . . . . . . G G G G G    
848	. . . . . . . . . . . . A A A A A    
849	. . . . . . . . . . . . C C C C C    
850	. . . . . . . . . . . . A A A A A    
851	. . . . . . . . . . . . G G G G G    
852	. . . . . . . . . . . . A A A A A    
853	. . . . . . . . . . . . A A A A A    
854	. . . . . . . . . . . . C C C C C    
855	. . . . . . . . . . . . A A A A A    
856	. . . . . . . . . . . . G G G G G    
857	. . . . . . . . . . . . A A A A A    
858	. . . . . . . . . . . . C C C C C    
859	. . . . . . . . . . . . A A A A A    
860	. . . . . . . . . . . . A A A A A    
861	. . . . . . . . . . . . A A A A A    
862	. . . . . . . . . . . . C C C C C    
863	. . . . . . . . . . . . C C C C C    
864	. . . . . . . . . . . . A A A A A    
865	. . . . . . . . . . . . A A A A A    
866	. . . . . . . . . . . . T T T T T    
867	. . . . . . . . . . . . A A A A A    
868	. . . . . . . . . . . . A A A A A    
869	. . . . . . . . . . . . A A A A A    
870	. . . . . . . . . . . . C C C C C    
871	. . . . . . . . . . . . A A A A A    
872	. . . . . . . . . . . . A A A A A    
873	. . . . . . . . . . . . G G G G G    
874	. . . . . . . . . . . . C C C C C    
875	. . . . . . . . . . . . C C C C C    
876	. . . . . . . . . . . . T T T T T    
877	. . . . . . . . . . . . C C C C C    
878	. . . . . . . . . . . . T T T T T    
879	. . . . . . . . . . . . C C C C C    
880	. . . . . . . . . . . . T T T T T    
881	. . . . . . . . . . . . G G G G G    
882	. . . . . . . . . . . . G G G G G    
883	. . . . . . . . . . . . G G G G G    
884	. . . . . . . . . . . . A A A A A    
885	. . . . . . . . . . . . A A A A A    
886	. . . . . . . . . . . . T T T T T    
887	. . . . . . . . . . . . . A A A A    
888	. . . . . . . . . . . . . G G G G    
889	. . . . . . . . . . . . . A A A A    
890	. . . . . . . . . . . . . A A A A    
891	. . . . . . . . . . . . . G G G G    
892	. . . . . . . . . . . . . A A A A    
893	. . . . . . . . . . . . . A A A A    
894	. . . . . . . . . . . . . T T T T    
895	. . . . . . . . . . . . . C C C C    
896	. . . . . . . . . . . . . T T T T    
897	. . . . . . . . . . . . . C C C C    
898	. . . . . . . . . . . . . T T T T    
899	. . . . . . . . . . . . . C C C C    
900	. . . . . . . . . . . . . C C C C    
901	. . . . . . . . . . . . . T T T T    
902	. . . . . . . . . . . . . T T T T    
903	. . . . . . . . . . . . . T T T T    
904	. . . . . . . . . . . . . C C C C    
905	. . . . . . . . . . . . . A A A A    
906	. . . . . . . . . . . . . C C C C    
907	. . . . . . . . . . . . . T T T T    
908	. . . . . . . . . . . . . C C C C    
909	. . . . . . . . . . . . . A A A A    
910	. . . . . . . . . . . . . A A A A    
911	. . . . . . . . . . . . . C C C C    
912	. . . . . . . . . . . . . C C C C    
913	. . . . . . . . . . . . . A A A A    
914	. . . . . . . . . . . . . T T T T    
915	. . . . . . . . . . . . . G G G G    
916	. . . . . . . . . . . . . A A A A    
917	. . . . . . . . . . . . . A A A A    
918	. . . . . . . . . . . . . G G G G    
919	. . . . . . . . . . . . . A A A A    
920	. . . . . . . . . . . . . T T T T    
921	. . . . . . . . . . . . . A A A A    
922	. . . . . . . . . . . . . A A A A    
923	. . . . . . . . . . . . . A A A A    
924	. . . . . . . . . . . . . A A A A    
925	. . . . . . . . . . . . . A A A A    
926	. . . . . . . . . . . . . T T T T    
927	. . . . . . . . . . . . . A A A A    
928	. . . . . . . . . . . . . A A A A    
929	. . . . . . . . . . . . . T T T T    
930	. . . . . . . . . . . . . G G G G    
931	. . . . . . . . . . . . . A A A A    
932	. . . . . . . . . . . . . A A A A    
933	. . . . . . . . . . . . . T T T T    
934	. . . . . . . . . . . . . A A A A    
935	. . . . . . . . . . . . . C C C C    
936	. . . . . . . . . . . . . T T T T    
937	. . . . . . . . . . . . . C C C C    
938	. . . . . . . . . . . . . A A A A    
939	. . . . . . . . . . . . . G G G G    
940	. . . . . . . . . . . . . G G G G    
941	. . . . . . . . . . . . . C C C C    
942	. . . . . . . . . . . . . T T T T    
943	. . . . . . . . . . . . . A A A A    
944	. . . . . . . . . . . . . T T T T    
945	. . . . . . . . . . . . . C C C C    
946	. . . . . . . . . . . . . A A A A    
947	. . . . . . . . . . . . . A A A A    
948	. . . . . . . . . . . . . C C C C    
949	. . . . . . . . . . . . . A A A A    
950	. . . . . . . . . . . . . T T T T    
951	. . . . . . . . . . . . . C C C C    
952	. . . . . . . . . . . . . T T T T    
953	. . . . . . . . . . . . . A A A A    
954	. . . . . . . . . . . . . C C C C    
955	. . . . . . . . . . . . . A A A A    
956	. . . . . . . . . . . . . C C C C    
957	. . . . . . . . . . . . . A A A A    
958	. . . . . . . . . . . . . G G G G    
959	. . . . . . . . . . . . . C C C C    
960	. . . . . . . . . . . . . C C C C    
961	. . . . . . . . . . . . . A A A A    
962	. . . . . . . . . . . . . T T T T    
963	. . . . . . . . . . . . . A A A A    
964	. . . . . . . . . . . . . C C C C    
965	. . . . . . . . . . . . . A A A A    
966	. . . . . . . . . . . . . G G G G    
967	. . . . . . . . . . . . . A A A A    
968	. . . . . . . . . . . . . A A A A    
969	. . . . . . . . . . . . . C C C C    
970	. . . . . . . . . . . . . A A A A    
971	. . . . . . . . . . . . . G G G G    
972	. . . . . . . . . . . . . A A A A    
973	. . . . . . . . . . . . . C C C C    
974	. . . . . . . . . . . . . C C C C    
975	. . . . . . . . . . . . . C A A A    
976	. . . . . . . . . . . . . . A A A    
977	. . . . . . . . . . . . . . T T T    
978	. . . . . . . . . . . . . . G G G    
979	. . . . . . . . . . . . . . A A A    
980	. . . . . . . . . . . . . . T T T    
981	. . . . . . . . . . . . . . A A A    
982	. . . . . . . . . . . . . . T T T    
983	. . . . . . . . . . . . . . A A A    
984	. . . . . . . . . . . . . . A A A    
985	. . . . . . . . . . . . . . C C C    
986	. . . . . . . . . . . . . . T T T    
987	. . . . . . . . . . . . . . G G G    
988	. . . . . . . . . . . . . . G G G    
989	. . . . . . . . . . . . . . G G G    
990	. . . . . . . . . . . . . . A A A    
991	. . . . . . . . . . . . . . T T T    
992	. . . . . . . . . . . . . . A A A    
993	. . . . . . . . . . . . . . T T T    
994	. . . . . . . . . . . . . . A A A    
995	. . . . . . . . . . . . . . A A A    
996	. . . . . . . . . . . . . . C C C    
997	. . . . . . . . . . . . . . C C C    
998	. . . . . . . . . . . . . . A A A    
999	. . . . . . . . . . . . . . A A A    
1000	. . . . . . . . . . . . . . T T T    
1001	. . . . . . . . . . . . . . G G G    
1002	. . . . . . . . . . . . . . A A A    
1003	. . . . . . . . . . . . . . T T T    
1004	. . . . . . . . . . . . . . A A A    
1005	. . . . . . . . . . . . . . T T T    
1006	. . . . . . . . . . . . . . C C C    
1007	. . . . . . . . . . . . . . T T T    
1008	. . . . . . . . . . . . . . C C C    
1009	. . . . . . . . . . . . . . A A A    
1010	. . . . . . . . . . . . . . T T T    
1011	. . . . . . . . . . . . . . G G G    
1012	. . . . . . . . . . . . . . T T T    
1013	. . . . . . . . . . . . . . T T T    
1014	. . . . . . . . . . . . . . C C C    
1015	. . . . . . . . . . . . . . T T T    
1016	. . . . . . . . . . . . . . G G G    
1017	. . . . . . . . . . . . . . A A A    
1018	. . . . . . . . . . . . . . A A A    
1019	. . . . . . . . . . . . . . G G G    
1020	. . . . . . . . . . . . . . G G G    
1021	. . . . . . . . . . . . . . A A A    
1022	. . . . . . . . . . . . . . G G G    
1023	. . . . . . . . . . . . . . T T T    
1024	. . . . . . . . . . . . . . G G G    
1025	. . . . . . . . . . . . . . T T T    
1026	. . . . . . . . . . . . . . T T T    
1027	. . . . . . . . . . . . . . T T T    
1028	. . . . . . . . . . . . . . G G G    
1029	. . . . . . . . . . . . . . G G G    
1030	. . . . . . . . . . . . . . A A A    
1031	. . . . . . . . . . . . . . G G G    
1032	. . . . . . . . . . . . . . A A A    
1033	. . . . . . . . . . . . . . A A A    
1034	. . . . . . . . . . . . . . A A A    
1035	. . . . . . . . . . . . . . G G G    
1036	. . . . . . . . . . . . . . A A A    
1037	. . . . . . . . . . . . . . G G G    
1038	. . . . . . . . . . . . . . G G G    
1039	. . . . . . . . . . . . . . G G G    
1040	. . . . . . . . . . . . . . A A A    
1041	. . . . . . . . . . . . . . C C C    
1042	. . . . . . . . . . . . . . T T T    
1043	. . . . . . . . . . . . . . A A A    
1044	. . . . . . . . . . . . . . C C C    
1045	. . . . . . . . . . . . . . T T T    
1046	. . . . . . . . . . . . . . G G G    
1047	. . . . . . . . . . . . . . G G G    
1048	. . . . . . . . . . . . . . A A A    
1049	. . . . . . . . . . . . . . G G G    
1050	. . . . . . . . . . . . . . T T T    
1051	. . . . . . . . . . . . . . G G G    
1052	. . . . . . . . . . . . . . G G G    
1053	. . . . . . . . . . . . . . T T T    
1054	. . . . . . . . . . . . . . T T T    
1055	. . . . . . . . . . . . . . C C C    
1056	. . . . . . . . . . . . . . A A A    
1057	. . . . . . . . . . . . . . C C C    
1058	. . . . . . . . . . . . . . T T T    
1059	. . . . . . . . . . . . . . T T T    
1060	. . . . . . . . . . . . . . T T T    
1061	. . . . . . . . . . . . . . A A A    
1062	. . . . . . . . . . . . . . G A A    
1063	. . . . . . . . . . . . . . T T T    
1064	. . . . . . . . . . . . . . G G G    
1065	. . . . . . . . . . . . . . C C C    
1066	. . . . . . . . . . . . . . A A A    
1067	. . . . . . . . . . . . . . C C C    
1068	. . . . . . . . . . . . . . A A A    
1069	. . . . . . . . . . . . . . G G G    
1070	. . . . . . . . . . . . . . C C C    
1071	. . . . . . . . . . . . . . A A A    
1072	. . . . . . . . . . . . . . T T T    
1073	. . . . . . . . . . . . . . C C C    
1074	. . . . . . . . . . . . . . A A A    
1075	. . . . . . . . . . . . . . A A A    
1076	. . . . . . . . . . . . . . C C C    
1077	. . . . . . . . . . . . . . T T T    
1078	. . . . . . . . . . . . . . T T T    
1079	. . . . . . . . . . . . . . C C C    
1080	. . . . . . . . . . . . . . A A A    
1081	. . . . . . . . . . . . . . A A A    
1082	. . . . . . . . . . . . . . A A A    
1083	. . . . . . . . . . . . . . C C C    
1084	. . . . . . . . . . . . . . A A A    
1085	. . . . . . . . . . . . . . G G G    
1086	. . . . . . . . . . . . . . A A A    
1087	. . . . . . . . . . . . . . C C C    
1088	. . . . . . . . . . . . . . A A A    
1089	. . . . . . . . . . . . . . G G G    
1090	. . . . . . . . . . . . . . A A A    
1091	. . . . . . . . . . . . . . A A A    
1092	. . . . . . . . . . . . . . T T T    
1093	. . . . . . . . . . . . . . C C C    
1094	. . . . . . . . . . . . . . C C C    
1095	. . . . . . . . . . . . . . T T T    
1096	. . . . . . . . . . . . . . C C C    
1097	. . . . . . . . . . . . . . C C C    
1098	. . . . . . . . . . . . . . T T T    
1099	. . . . . . . . . . . . . . T T T    
1100	. . . . . . . . . . . . . . T T T    
1101	. . . . . . . . . . . . . . T T T    
1102	. . . . . . . . . . . . . . T T T    
1103	. . . . . . . . . . . . . . C C C    
1104	. . . . . . . . . . . . . . A A A    
1105	. . . . . . . . . . . . . . T T T    
1106	. . . . . . . . . . . . . . C C C    
1107	. . . . . . . . . . . . . . A A A    
1108	. . . . . . . . . . . . . . G G G    
1109	. . . . . . . . . . . . . . T T T    
1110	. . . . . . . . . . . . . . T T T    
1111	. . . . . . . . . . . . . . C C C    
1112	. . . . . . . . . . . . . . A A A    
1113	. . . . . . . . . . . . . . T T T    
1114	. . . . . . . . . . . . . . C C C    
1115	. . . . . . . . . . . . . . C C C    
1116	. . . . . . . . . . . . . . A A A    
1117	. . . . . . . . . . . . . . C C C    
1118	. . . . . . . . . . . . . . T T T    
1119	. . . . . . . . . . . . . . C C C    
1120	. . . . . . . . . . . . . . A A A    
1121	. . . . . . . . . . . . . . C C C    
1122	. . . . . . . . . . . . . . T T T    
1123	. . . . . . . . . . . . . . G G G    
1124	. . . . . . . . . . . . . . G G G    
1125	. . . . . . . . . . . . . . A A A    
1126	. . . . . . . . . . . . . . G G G    
1127	. . . . . . . . . . . . . . T T T    
1128	. . . . . . . . . . . . . . G G G    
1129	. . . . . . . . . . . . . . T T T    
1130	. . . . . . . . . . . . . . A A A    
1131	. . . . . . . . . . . . . . A A A    
1132	. . . . . . . . . . . . . . C C C    
1133	. . . . . . . . . . . . . . C C C    
1134	. . . . . . . . . . . . . . C G G    
1135	. . . . . . . . . . . . . . . T T    
1136	. . . . . . . . . . . . . . . G G    
1137	. . . . . . . . . . . . . . . G G    
1138	. . . . . . . . . . . . . . . A A    
1139	. . . . . . . . . . . . . . . C C    
1140	. . . . . . . . . . . . . . . T T    
1141	. . . . . . . . . . . . . . . T T    
1142	. . . . . . . . . . . . . . . C C    
1143	. . . . . . . . . . . . . . . T T    
1144	. . . . . . . . . . . . . . . A A    
1145	. . . . . . . . . . . . . . . T T    
1146	. . . . . . . . . . . . . . . A A    
1147	. . . . . . . . . . . . . . . C C    
1148	. . . . . . . . . . . . . . . A A    
1149	. . . . . . . . . . . . . . . C C    
1150	. . . . . . . . . . . . . . . A A    
1151	. . . . . . . . . . . . . . . C C    
1152	. . . . . . . . . . . . . . . A A    
1153	. . . . . . . . . . . . . . . G G    
1154	. . . . . . . . . . . . . . . A A    
1155	. . . . . . . . . . . . . . . T T    
1156	. . . . . . . . . . . . . . . G G    
1157	. . . . . . . . . . . . . . . C C    
1158	. . . . . . . . . . . . . . . C C    
1159	. . . . . . . . . . . . . . . T T    
1160	. . . . . . . . . . . . . . . A A    
1161	. . . . . . . . . . . . . . . G G    
1162	. . . . . . . . . . . . . . . A A    
1163	. . . . . . . . . . . . . . . C C    
1164	. . . . . . . . . . . . . . . C C    
1165	. . . . . . . . . . . . . . . A A    
1166	. . . . . . . . . . . . . . . G G    
1167	. . . . . . . . . . . . . . . A A    
1168	. . . . . . . . . . . . . . . G G    
1169	. . . . . . . . . . . . . . . T T    
1170	. . . . . . . . . . . . . . . A A    
1171	. . . . . . . . . . . . . . . G G    
1172	. . . . . . . . . . . . . . . T T    
1173	. . . . . . . . . . . . . . . A A    
1174	. . . . . . . . . . . . . . . T T    
1175	. . . . . . . . . . . . . . . C C    
1176	. . . . . . . . . . . . . . . C C    
1177	. . . . . . . . . . . . . . . A A    
1178	. . . . . . . . . . . . . . . G G    
1179	. . . . . . . . . . . . . . . C C    
1180	. . . . . . . . . . . . . . . T T    
1181	. . . . . . . . . . . . . . . G G    
1182	. . . . . . . . . . . . . . . A A    
1183	. . . . . . . . . . . . . . . T T    
1184	. . . . . . . . . . . . . . . C C    
1185	. . . . . . . . . . . . . . . T T    
1186	. . . . . . . . . . . . . . . G G    
1187	. . . . . . . . . . . . . . . C C    
1188	. . . . . . . . . . . . . . . A A    
1189	. . . . . . . . . . . . . . . A A    
1190	. . . . . . . . . . . . . . . G G    
1191	. . . . . . . . . . . . . . . C C    
1192	. . . . . . . . . . . . . . . T T    
1193	. . . . . . . . . . . . . . . T T    
1194	. . . . . . . . . . . . . . . T T    
1195	. . . . . . . . . . . . . . . G G    
1196	. . . . . . . . . . . . . . . T T    
1197	. . . . . . . . . . . . . . . C C    
1198	. . . . . . . . . . . . . . . A A    
1199	. . . . . . . . . . . . . . . T T    
1200	. . . . . . . . . . . . . . . G G    
1201	. . . . . . . . . . . . . . . C C    
1202	. . . . . . . . . . . . . . . A A    
1203	. . . . . . . . . . . . . . . C C    
1204	. . . . . . . . . . . . . . . G G    
1205	. . . . . . . . . . . . . . . T T    
1206	. . . . . . . . . . . . . . . A A    
1207	. . . . . . . . . . . . . . . G G    
1208	. . . . . . . . . . . . . . . A A    
1209	. . . . . . . . . . . . . . . G G    
1210	. . . . . . . . . . . . . . . T T    
1211	. . . . . . . . . . . . . . . A A    
1212	. . . . . . . . . . . . . . . C C    
1213	. . . . . . . . . . . . . . . A A    
1214	. . . . . . . . . . . . . . . T T    
1215	. . . . . . . . . . . . . . . C C    
1216	. . . . . . . . . . . . . . . T T    
1217	. . . . . . . . . . . . . . . T T    
1218	. . . . . . . . . . . . . . . G G    
1219	. . . . . . . . . . . . . . . A A    
1220	. . . . . . . . . . . . . . . T T    
1221	. . . . . . . . . . . . . . . A A    
1222	. . . . . . . . . . . . . . . T T    
1223	. . . . . . . . . . . . . . . A A    
1224	. . . . . . . . . . . . . . . A A    
1225	. . . . . . . . . . . . . . . T T    
1226	. . . . . . . . . . . . . . . C C    
1227	. . . . . . . . . . . . . . . C C    
1228	. . . . . . . . . . . . . . . T T    
1229	. . . . . . . . . . . . . . . G G    
1230	. . . . . . . . . . . . . . . A A    
1231	. . . . . . . . . . . . . . . A A    
1232	. . . . . . . . . . . . . . . T T    
1233	. . . . . . . . . . . . . . . A A    
1234	. . . . . . . . . . . . . . . G G    
1235	. . . . . . . . . . . . . . . T T    
1236	. . . . . . . . . . . . . . . A A    
1237	. . . . . . . . . . . . . . . C C    
1238	. . . . . . . . . . . . . . . T T    
1239	. . . . . . . . . . . . . . . A A    
1240	. . . . . . . . . . . . . . . A A    
1241	. . . . . . . . . . . . . . . C C    
1242	. . . . . . . . . . . . . . . T T    
1243	. . . . . . . . . . . . . . . G G    
1244	. . . . . . . . . . . . . . . T T    
1245	. . . . . . . . . . . . . . . A A    
1246	. . . . . . . . . . . . . . . A A    
1247	. . . . . . . . . . . . . . . C C    
1248	. . . . . . . . . . . . . . . T T    
1249	. . . . . . . . . . . . . . . G G    
1250	. . . . . . . . . . . . . . . A A    
1251	. . . . . . . . . . . . . . . G G    
1252	. . . . . . . . . . . . . . . C G    
1253	. . . . . . . . . . . . . . . . T    
1254	. . . . . . . . . . . . . . . . A    
1255	. . . . . . . . . . . . . . . . T    
1256	. . . . . . . . . . . . . . . . T    
1257	. . . . . . . . . . . . . . . . T    
1258	. . . . . . . . . . . . . . . . C    
1259	. . . . . . . . . . . . . . . . T    
1260	. . . . . . . . . . . . . . . . C    
1261	. . . . . . . . . . . . . . . . C    
1262	. . . . . . . . . . . . . . . . A    
1263	. . . . . . . . . . . . . . . . C    
1264	. . . . . . . . . . . . . . . . C    
1265	. . . . . . . . . . . . . . . . A    
1266	. . . . . . . . . . . . . . . . G    
1267	. . . . . . . . . . . . . . . . A    
1268	. . . . . . . . . . . . . . . . A    
1269	. . . . . . . . . . . . . . . . A    
1270	. . . . . . . . . . . . . . . . A    
1271	. . . . . . . . . . . . . . . . C    
1272	. . . . . . . . . . . . . . . . C    
1273	. . . . . . . . . . . . . . . . C    
1274	. . . . . . . . . . . . . . . . C    
1275	. . . . . . . . . . . . . . . . C    
1276	. . . . . . . . . . . . . . . . C    
1277	. . . . . . . . . . . . . . . . C    
1278	. . . . . . . . . . . . . . . . C
```
